# Autistic women’s experiences of self-compassion after receiving their diagnosis in adulthood

**DOI:** 10.1177/13623613221136752

**Published:** 2022-11-14

**Authors:** Rosemarie B Wilson, Andrew R Thompson, Georgina Rowse, Richard Smith, Amber-Sophie Dugdale, Megan Freeth

**Affiliations:** 1 University of Sheffield, UK; 2 Cardiff University, UK; 3 Derbyshire Community Health Services, NHS Foundation Trust, UK

**Keywords:** adults, autistic women, diagnosis, health services, interpretative phenomenological analysis, lived experience, qualitative research, self-compassion

## Abstract

Knowledge of autistic individuals’ experiences of self-compassion is very limited. This study investigated autistic women’s experiences of self-compassion after receiving their diagnosis in adulthood. Eleven autistic women completed semi-structured interviews analysed using interpretative phenomenological analysis. Three super-ordinate themes were identified: ‘Disconnect between the autistic self and experience of societal expectations’ (the burden of conformity; autism is misunderstood; social challenges; mental health impact); ‘Unmasking: the process of self-understanding’ (autonomy and self-compassion; validation and grief) and ‘Impact on relationships’ (diagnosis disclosure dilemmas; connection and understanding). Frustration with society’s misconceptions of autism and unhelpful thinking styles were presented as barriers to self-compassion. Most participants reported that their diagnosis had led to the development of a greater sense of self-understanding, which facilitated self-compassion. Some participants suggested their own increased understanding of autism facilitated their compassion towards others. Findings from this study have clinical implications for increasing understanding about autistic women’s experiences of self-compassion and possibly ways to facilitate its development, to enhance well-being.

**Lay abstract:**

Knowledge of autistic individuals’ experiences of self-compassion is very limited. This study investigated autistic women’s experiences of self-compassion after receiving their diagnosis in adulthood. Eleven autistic women were interviewed about their experiences of receiving their diagnosis in adulthood and their experiences of self-compassion. Systematic analysis of the interview transcripts revealed common themes in the participants’ experiences. Participants reported that their autism diagnosis helped them to better understand themselves, particularly when reflecting on problematic past experiences. After receiving an autism diagnosis, participants described being able to relate to themselves with greater self-kindness compared to previous self-criticism; this included allowing themselves to assert their needs and engage in self-care activities. Participants spoke about having difficult social experiences, including feeling pressure to conform to expectations in society and often feeling misunderstood. The findings highlight the barriers autistic women face obtaining their diagnoses and demonstrate the need for autism training for professionals to support early identification. Findings from this study suggest that interventions aimed at developing self-compassion could support and enhance autistic women’s well-being.

## Introduction

Research investigating the experience of being autistic^1^ has been emerging in recent years (DePape & Lindsay, 2016). Findings suggest that autistic girls and women experience significant barriers to receiving an autism diagnosis compared with boys and men (Lewis, 2017). Autistic girls and women also tend to be diagnosed later in life than boys and men (Begeer et al., 2013; Siklos & Kerns, 2007). A barrier to diagnosis can be the use of compensatory strategies to ‘mask’ or ‘camouflage’ difficulties (Hull et al., 2017). A recent systematic review of studies considering camouflaging in all sexes and genders revealed that autistic females and girls/women demonstrate more camouflaging than autistic males and boys/men (Cook et al., 2021). These compensatory strategies can take significant cognitive and emotional effort and negatively impact mental health (Bargiela et al., 2016).

Mental health issues are common in autistic people, with one study reporting that 79% of autistic adults met diagnostic criteria for a mental health condition (Lever & Geurts, 2016). Research suggests autism acceptance contributes to better mental health in autistic individuals. Specifically, autism acceptance from external sources and personal acceptance significantly reduced depression (Cage et al., 2018). There is also the potential for diagnostic overshadowing of an autism diagnosis, whereby co-occurring mental health conditions are neglected which can have negative consequences for the individual (Matson & Williams, 2013). Research suggests autistic girls’/women’s needs are under recognised (Gould, 2017) and their support needs are often misunderstood and misattributed to different diagnoses (Attwood, 2007).

Self-compassion is defined by Neff (2003) as comprising three interrelated elements: ‘self-kindness versus self-judgement, common humanity versus isolation and mindfulness versus over-identification’. Self-compassion research is rapidly growing due to its strong link with mental health and psychological benefits (Barnard & Curry, 2011; Gilbert, 2009). Previous research suggests neurotypical women have lower levels of self-compassion than men (Yarnell et al., 2015). There is limited research exploring self-compassion in relation to autism. A small number of studies have explored self-compassion in parents of autistic children (Neff & Faso, 2015; Wong et al., 2016). Galvin et al. (2021) examined whether self-compassion is associated with autistic traits in a sample without a clinical autism diagnosis, finding evidence that self-compassion is a partial mediator in the relationship between increased autistic traits and reduced depressive/anxiety symptomology. Based on these findings, Galvin et al. (2021) suggest self-compassion could be a target for clinical intervention for people with high autistic traits experiencing depression and/or anxiety. It is possible that autistic women and women with subclinical autistic traits may be more vulnerable to experiencing reduced self-compassion as they are more likely to internalise their difficulties (Bargiela et al., 2016; Mandy et al., 2012; Scherff et al., 2014). In a recent qualitative study, several autistic women reported that receiving their autism diagnosis in adulthood supported a transition from self-criticism to self-compassion (Leedham et al., 2020). Building on Leedham et al’.s (2020) findings, the current research aims to explore autistic women’s experiences of being compassionate towards themselves and how this is perceived to relate to their wider experiences.

Research exploring autistic girls’/women’s experiences of diagnosis is developing (Bargiela et al., 2016). Several autistic women in Leedham et al.’s (2020) study reported that receiving their autism diagnosis increased their sense of agency and acceptance of the self. Other research reported that diagnosis was linked to feelings of relief, providing answers to questions participants had about themselves (Punshon et al., 2009). Comparatively, difficult feelings associated with diagnosis, such as grief, are also reported in the literature (Leedham et al., 2020; Portway & Johnson, 2005). This study aims to add to the growing area of research exploring autistic women’s experiences and specifically aims to improve understanding of experiences relating to self-compassion.

There is no current research specifically exploring autistic individuals’ experiences of self-compassion. Given the existing research reporting the relationship between self-compassion and mental health, this study has clinical implications for increasing understandings about autistic women’s experiences of self-compassion and possibly ways to facilitate its development. As well as general consideration of self-compassion, this study specifically focuses on how diagnosis may influence self-compassion. This will also contribute to understanding the value of the diagnosis in the autistic community and the role that self-compassion may play.

## Method

### Methodological approach

This study employed interpretative phenomenological analysis (IPA: Smith et al., 2009). IPA is a rigorous qualitative approach, and in this study, each participant engaged in a semi-structured interview, and the interview transcripts were subsequently analysed in accordance with techniques specified within the approach. IPA facilitates the exploration of personal meanings and subjective interpretations, in this case specifically focussing on individuals’ lived experiences of self-compassion, and how this might relate to the experience of gaining an autism diagnosis in adulthood. IPA is now an established method for gaining close and detailed understandings of autistic people’s experiences (Griffith et al., 2012; MacLeod, 2019; Treweek et al., 2019; D. Ward & Webster, 2018), and it has been argued that using IPA in autism research goes some way towards alleviating the ‘double empathy problem’ (Milton, 2012), which can affect other approaches that have been used in autism research (Howard et al., 2019). The ‘double empathy problem’ (Milton, 2012) describes the possible mutual misunderstandings between neurotypical and autistic people, potentially threatening the authenticity of qualitative data.

Ethical approval was obtained via the Integrated Research Application System (IRAS project ID: 275864) and from the local NHS Foundation Trust for governance.

### Participants

Eleven participants were recruited via purposive sampling involving an NHS diagnostic service and a research centre website. Participants self-identified as to whether they met the study requirements. Inclusion criteria were as follows: clinical autism diagnosis received after their 18th birthday, cis gender female. Participants were excluded if they lacked the capacity to provide consent, were unable to speak English and did not have access to the Internet (due to COVID-19 restrictions, consent was obtained electronically). See Table 1 for demographic information. All names are pseudonyms to protect confidentiality. Some participants reported additional diagnoses including dyspraxia, depression and anxiety.

**Table 1.** Participant demographics.

Participant	Age at interview*	Time since diagnosis	Diagnosis	Ethnicity
Jessica	40–44	>5 years	ASC as seen in women and girls	White British
Stacey	35–39	<6 months	ASD	White British
Tara	55–59	>5 years	AS	White
Melissa	45–49	>5 years	Autism	White British
Karen	65–69	6–12 months	ASD	White British
Angela	60–64	6–12 months	ASD	White British
Debby	55–59	1–5 years	ASD	White British
Juliet	35–39	<6 months	ASD	White British
Natasha	35–39	<6 months	ASD (formally AS)	White British
Louise	35–39	<6 months	ASD	White British
Sue	35–39	<6 months	ASD	White British

Note. ASC: autism spectrum condition; ASD: autism spectrum disorder (American Psychiatric Association, 2013); AS: Asperger’s syndrome, diagnostic description based on previous diagnostic criteria.

* Exact ages are not reported to protect confidentiality.

### Procedure

Staff at the NHS service provided research information flyers at diagnosis follow-up, and research information was also displayed on the research laboratory website. The self-compassion focus of the study was clearly advertised by the study title and project summary information. Participants expressed their interest by recording their contact details on a secure online form. A participant information sheet and a consent form were sent to participants that were eligible to take part. Participants were informed that the aim of the study was to investigate ‘autistic women’s experiences of self-compassion and whether receiving the diagnosis in adulthood has influenced experiences of self-compassion’. Participants were also provided with the interview schedule in advance if requested. The interview schedule was designed in line with methodological guidance and based on the aims of study (Larkin & Thompson, 2012: see Table 2). Prompts were utilised to request further information and clarification. Due to COVID-19 restrictions, interviews were conducted via video call or telephone, depending on the participant’s preference. Audio from the interviews was recorded via an encrypted smart tablet. Interviews ranged between 37 and 96 min. At the start of each interview, the limits of confidentiality were discussed, and participants were debriefed at the end of the interviews.

**Table 2.** Summary of semi-structured interview schedule main questions.

Question number	Question wording
1.	What was the exact diagnosis you were given?
2.	What does (insert the term the participant uses) mean to you? (or) How would you describe autism (or alternative preferred term) to someone?
3.	What effect has your diagnosis has on your life?
4.	What effect has receiving the diagnosis had on how you feel about yourself?
5.	How do you act towards yourself in difficult times?
6.	Are there certain things that make it easier (or harder) to be kinder or more forgiving towards yourself?
7.	How do you feel the way you think and behave towards yourself compares with what other people experience?
8.	Is there anything else you would like to share about how you feel towards yourself self receiving the diagnosis of (preferred term)?

### Analysis

Transcripts were analysed in accordance with IPA guidance (Larkin & Thompson, 2012; Smith et al., 2009). The interviews were transcribed verbatim. Initially, transcripts were read while listening to audio recordings to ensure accuracy of transcribing and familiarity with the original data. During this early stage of analysis, exploratory comments were recorded on the right-hand margin. Transcripts were then read again, and ‘line by line’ coding of the data was conducted, recording descriptive, linguistic and conceptual comments in the right-hand margin. Next, while re-reading the transcripts, tentative emerging themes and possible interpretations were recorded on the left margin of transcripts. Then, individual data were organised into themes, creating conceptual maps using online software, Mind Manager. Comparisons of data across the transcripts identified overlapping concepts and exceptions. Clusters of themes were developed alongside psychological theory and research while remaining grounded in the participant’s accounts. Clusters established a hierarchical relationship of super-ordinate and sub-ordinate theme categories. Three of the authors and an independent researcher completed an audit of each stage of analysis for three transcripts to ensure that the findings were traceable back to the data and that it was evident that the analytic process had been systematic (Larkin & Thompson, 2012). The first author kept a reflective log throughout the course of the study. This was used to note the influence of both theoretical experience and personal experience on the interpretations that emerged during the analysis. This process was used not to guard against ‘bias’ but rather to ensure that reflexivity (Shaw, 2010) was objectively considered and that the interpretations underpinning the themes could be intellectually scrutinised.

### Community involvement statement

The lead researcher attended a discussion group at a local NHS autism diagnostic service to gain feedback on the proposed research. The group included nine autistic adults, both men and women, all diagnosed in adulthood. The group feedback informed the terminology used on the participant information sheets and the interview schedule. Two autistic adults were contacted from the research laboratory database, and they also reviewed the interview schedule and participant-facing information. These consultations provided advice regarding the accessibility of the language and terminology, consideration of ethical and methodological issues.

## Results

Analysis of the data produced three super-ordinate themes and several sub-themes; see Table 3 for details. Participants’ quotes are provided to illustrate each theme; missing data are represented by the use of ‘. . ’, and additional explanation of points are included within ‘[ ]’. The themes do not provide an exhaustive account of participants’ experiences; they provide an interpretation of several accounts. Pseudonyms are used to protect participant’s anonymity.

**Table 3.** Super-ordinate themes and sub-themes.

Super-ordinate themes	Sub-themes
Disconnect between the autistic self and experience of societal expectations	The burden of conformity
	Autism is misunderstood
	Social challenges
	Mental health impact
Unmasking: the process of self-understanding	Autonomy and self-compassion
	Validation and grief
Impact on relationships	Diagnosis disclosure dilemmas
	Connection and understanding

### Theme 1 – disconnect between the autistic self and experience of societal expectations

This super-ordinate theme explores participants’ experiences of feeling different from society’s neurotypical expectations, creating feelings of rejection, frustration and ‘*disconnect between me and society’* (Angela). Participants often reported feeling that autism is misunderstood and described common unhelpful stereotypes. All the participants recalled social challenges, including attempts to navigate social rules. Some participants reflected on the impacts of these experiences on their mental health.

#### The burden of conformity

Participants reflected on their autism diagnosis in comparison with societal norms and the pressure to conform to neurotypical expectations:

being left-handed in a right-handed world. . . I‘ve been in a neurotypical world and people have been trying to treat me and condition me as if I was neurotypical and I’M NOT! (Karen)

Karen suggests that she feels society wants to change or ‘*repair*’ (Angela) her to be more neurotypical. ‘*Treat*’ suggests that there is perception that something is wrong, requiring intervention, echoing traditional medical model narratives of autism. Other participants shared similar views stating they were often ‘*made to feel less*’ (Debby) or ‘*broken*’ (Sue, Juliet, Karen). During the interviews, Juliet and Melissa also reflected on applied behavioural analysis (ABA), which controversially employs conditioning methods to change autistic children’s behaviour. Understandably, most participants expressed disappointment and feeling ‘*frustrated at the world at large*’ (Stacey) towards society’s intolerance of difference and various rules:

‘so many straight-jackets and so much rigidity, most of it unspoken’ (Angela). Angela’s description illustrates her perception of societal expectations as vague and restrictive. Participants also expressed feelings of confusion and injustice when reflecting on social rules. Reflections at the ‘world’ emphasise the intensity of these feelings, suggesting the experience is relentless with little reprieve from the reminder of their felt differences. These descriptions also position participants as separate from the world rather than a part of it. Similarly, Debby is the mother of three autistic children, and she described other people’s distress as ‘waves’ compared with ‘tsunamis’ which was her family experience. This comparison positions her emotional pain as more intense and distinct to her personal experience, increasing her sense of isolation. Participants’ descriptions of isolation and feeling different regarding their diagnosis potentially limit their resources for self-compassion.

#### Autism is misunderstood

Participants described an overwhelming sense of feeling misunderstood, underpinned by society’s neurotypical definitions and expectations of autism. For some participants, these feelings prompted their aspirations to advocate for inclusion. Participants reported wanting to improve other people’s understanding of autism by facilitating autism training (Juliet, Natasha and Melissa) and participating in research. Participants expressed their frustration when reflecting on misconceptions of autism, including assumptions about intellectual abilities and suggestions that autism only occurs in males. Many participants suggested the media’s portrayal of autism further perpetuates misunderstandings. Participants tended to be cautious in their responses when describing some of their experiences, not wanting to generalise to other autistic people, contrasting with how participants felt society often groups together their idiographic experiences.

A lot of people still think of autism as nonverbal, rocking in a corner. . . they tend to be less aware that somebody who looks normal and is articulate and well educated can still have (pause), have a spectrum disorder (Karen).

Two participants (Juliet & Melissa) specifically referred to ABA and invalidating descriptions they have heard expressed about the autistic community:

people who say they would abort their child if they were Autistic. . . people who are Autistic need to be more neurotypical and kind of backing the ABA therapy. . . I had this compassion for myself then I came across some of these comments and I just felt awful. (Juliet)

This quote links to participants’ reports of feeling rejected by not meeting society’s expectations, with the ultimate rejection to be excluded from existence. Understandably, hearing these extreme views is reported to impact Juliet’s self-compassion negatively. This illustrates how criticism from others can be internalised. Comparatively, most participants also referred to helpful resources from the autistic community (e.g. Temple Grandin) to challenge these misconceptions. Overall, participants felt inclusion could be improved and shared hopes for society to celebrate neurodiversity.

#### Social challenges

All participants reported social challenges, including experiences of victimisation, ‘*gaslighting*’ (Melissa & Karen) and interpersonal difficulties. Participants described the enormous effort required for social interactions and the necessity of self-care activities such as ‘*downtime*’ (Jessica) and *‘not doing’* (Tara) to ‘*recharge*’ (Sue, Karen) their depleted energy resources. Many participants described the challenges of social expectations and expressed confusion around social rules:

it felt like erm, other people had been given an instruction manual on how to navigate the world and I hadn’t been given that. (Stacey)

Despite the immense energy required for socialising, many participants described efforts to seek approval from others. Discussions illustrated how social interactions are internalised and influence participant’s experiences of self-compassion. Debby described how receiving compassion from others cultivates her self-compassion:

. . . it’s easier to be kind to myself and forgiving when I’ve had a more positive interactions.

Participants varied in their evaluations of social challenges they had experienced prior to receiving their autism diagnosis. Some suggested their diagnosis facilitated a shift from self-blame to anger towards people that had been unkind to them:

I just felt bad about myself at the time. . . now I feel good about myself and cross with them. (Karen).

In contrast, Tara described feeling ‘*more forgiving*’ of others, when reflecting on past social difficulties. She reported that receiving her autism diagnosis had prompted her to repair a relationship over 30 years after an argument. This suggested an increase in her compassion towards others post-diagnosis.

#### Mental health impact

Considering the sense of rejection from society and social challenges, unsurprisingly, most participants discussed experiencing mental health issues. *‘. . .depression and anxiety from feeling so different and that I didn’t understand the rest of the world*’. (Karen)

Karen’s description indicates elements of self-blame for not understanding the world and emphasises the sense of isolation. Participants also described feeling misunderstood by professionals and being misdiagnosed. Some participants reflected on the timing of their autism diagnosis, and if they had been diagnosed earlier in life, their mental health might be improved:

they diagnosed the social anxiety whereas I think it’s just a reaction to you know the stress of masking. . . apparently that’s quite common with autism that you get a lot of false diagnoses before you get the one that actually is you. (Angela)

This description challenges the medical model approach and illustrates the power dynamic between professionals imposing diagnostic labels, which contrasted with Angela’s understanding. Participants’ reports also highlighted the psychological impact of rejection and the unintended consequences of coping strategies such as masking.

you end up feeling like you, you’ve got either a deficiency or you’re broken in some way, shape or form. (Sue)

Pre-diagnosis, participants internalised the negative reactions from others, adding to their negative self-perception. This way of self-relating is linked to the ‘self-kindness versus self judgement’ element of Neff’s (2003) definition and presents participants’ self-criticism as a barrier to their self-compassion.

### Theme 2 – unmasking: the process of self-acceptance

This super-ordinate theme considers how participants’ autism diagnosis facilitated greater self-understanding and provided a new ‘*lens*’ (Debby & Jessica) to their difficult past experiences. All participants expressed a range of emotions about receiving their diagnosis, including feelings of validation and grief. Most participants suggested that their increased self-understanding had developed their self-acceptance, while also recognising the process of ‘*unlearning*’ (Melissa) and ‘*unmasking*’ (Sue) would take time due to receiving the diagnosis later in life.

#### Autonomy and self-compassion

Participants described their autism diagnosis to explain some of their difficult experiences, particularly when reflecting on challenges in the past. This new understanding transformed previous negative feelings of ‘*failure*’ (Stacey) and ‘*blame*’ (Natasha, Debby) and provided a new ‘*less critical*’ (Karen) view of themselves. For most participants, the diagnosis facilitated greater self-understanding and self-compassion:

. . . why am I like this’, this little voice told me and now it’s like I understand what I’m going through and I can sort of de-escalate it quicker. . . it’s like I’m accepting of who I am whereas before I wasn’t at all. (Juliet)

Participants referred to their sense of autonomy, suggesting that their diagnosis provided ‘*permission*’ (Jessica) to explain and ‘*a backing*’ (Natasha) to assert their needs; ‘*it’s just letting myself be me a bit more*’. (Stacey). Most participants reported setting boundaries for self-care post-diagnosis, particularly with social activities and needing ‘*downtime*’ (Jessica). Generally, learning to maintain boundaries was described as helpful; however; some participants expressed a concern that asserting their needs could limit their experiences:

I probably have been a bit kinder with myself, but again it’s striking that balance between being kind enough to yourself and not overindulging yourself. (Stacey).

Most participants reflected on the process of adjusting to the new experience of self-kindness post-diagnosis. Stacey’s quote also presents a common negative perception of self-compassion; fearing self-compassion is a weakness or self-indulgent. It is unsurprising that self-compassion may feel threatening when people have had limited experiences of compassion previously. This quote also alludes to participants’ commonly reported striving traits; it could be argued participants are striving to be accepted by society and also reflect their efforts to accept themselves post-diagnosis.

Increased self-understanding from the diagnosis prompted participants to research autism and recommended soothing items, for example weighted blankets. Other commonly reported self-care strategies included spending time in nature, sensory activities (touch and music) and having time alone, ‘*being on my own, being wrapped up*’. (Stacey)

Increased self-understanding allowed most participants to begin to reveal their authentic selves.

. . . it’s given me my identity back because that had just totally vanished from the amount of masking I were doing . . . (Sue)

This process suggests the diagnosis facilitated participant’s experiences of self-compassion, accepting their reality with kindness, rather than commonly report self-criticism pre-diagnosis.

#### Validation and grief

All the participants had requested their autism assessments, and initially, the diagnosis provided ‘*relief*’ (Melissa, Juliet, Sue & Karen) confirming their suspicions.

it felt vindicating, it felt affirming, it felt really good, that, that I wasn’t sort of erm mad or bad just, just different. . . it makes me feel a bit special I think (laugh), quite valuable and I think I see the skills that I have more than feeling a bit of a freak (Karen).

This quote illustrates how the diagnosis facilitated Karen to reframe her differences as ‘*skills*’, compared with feeling a ‘*freak*’. This description supports the suggestion the diagnosis facilitated participants’ self-compassion.

Over time, the initial relief and ‘*euphoria*’ (Debby) towards receiving the diagnosis were accompanied by conflicting feelings, highlighting the ‘*long process to unravel all the damage there’s been done from being undiagnosed’* (Melissa) and uncertainty for the future.

you know when you try on a new suit and you’re just getting comfortable in it and you’re still not quite comfortable, you’re not sure if it suits you or if it fits. (Jessica)

Jessica’s metaphor of ‘*a new suit*’ illustrates the process of adjustment, the initial discomfort and alludes to the formality of the diagnosis. Most participants described ‘*a grieving process’* (Debby) and sadness towards their late diagnosis. In comparison, Natasha wondered if she would be as successful in her employment if she had received her diagnosis earlier:

. . .would I have pushed myself to get to where I wanna be or where I am now had I had that [diagnosis].

Arguably, this links the participants’ striving traits with the experience of living with undiagnosed autism. Alternatively, this view may be more beneficial for Natasha’s self-preservation as ultimately; it is impossible to know how an earlier diagnosis may have impacted her life. Some participants described questioning themselves, reflecting on the permanence of the diagnosis, their initial expectations and occasional feelings of frustration:

I do sometimes question why I am autistic, sort of why me. (Tara)

### Theme 3 – impact on relationships

This super-ordinate theme explores the impact on participant’s relationships post-diagnosis, exploring reflections on diagnosis disclosure and shared experiences of autism.

#### Diagnosis disclosure dilemmas

Participants described unhelpful stereotypes and previously invalidating responses as barriers to their autism diagnosis disclosure. Participants reflected on the process of adjustment to disclosing their diagnosis:*‘I’m starting to accept myself so I feel comfortable saying it [diagnosis] now*’. (Juliet) Most participants described feeling hesitant about sharing their diagnosis with their employer and colleagues unless they require reasonable adjustments in the workplace. Sue was looking for work at the time of the interview and shared her dilemma about disclosing her diagnosis and suggested she was suspicious about future employer’s inclusivity.

. . .I’m not just going to be totally out and open with absolutely everyone straight away and go ‘I’m Autistic’ because they will think stereotypical things. . . I know legally in Disability Law you’re not meant to erm discriminate but how are they gonna prove that at the end of the day.

Participants reported supportive responses post-diagnosis disclosure from close friends and family but shared concerns about the impact of disclosure in professional settings.

. . .my family know and they’ve been amazing about it, erm and I’ve told some really close friends and they’ve all been great about it but professionally I’ve been really guarded because I don’t want anyone to judge me on that. . . (Jessica)

#### Connection and understanding

Participants suggested their shared experiences with other autistic people facilitate understanding and connection.

I do make an effort to see my friend I made through that autism group, it’s nice to talk about our experiences. . . what we’re finding out about ourselves and to be with someone that you’re not worried about being judged. (Sue)

Sue, Natasha and Angela shared that since their diagnosis they suspected that one of their parents was undiagnosed autistic. Their suspicions helped make sense of challenges in their relationships, arguably cultivating compassion for their parents. In addition, Louise, Natasha, Karen, Debby and Jessica reported that their children or siblings also had confirmed or suspected autism. Two participants reflected on their enhanced ability to understand their client’s needs in their employment working with autistic adults and children with additional needs.

. . . to me it just feels normal. . .it feels natural and I can help them, I don’t know what they are thinking but I can sense what they are wanting to do and feeling. . . if I hadn’t got autism I probably wouldn’t have been able to help them to that extent. . . (Louise)

In contrast, Debby shared that she continues to feel disconnected from peers post-diagnosis:

. . .this new life even though theoretically I’m better informed to find that tribe but I don’t always feel a part of it. . . (Debby).

This quote indicates that connection did not necessarily increase for all participants post-diagnosis.

## Discussion

This study aimed to investigate autistic women’s experiences of self-compassion and whether receiving their diagnosis in adulthood influenced their reflections on self-compassion. Three super-ordinate themes emerged: *‘Disconnect between the autistic self and experience of societal expectations’*, ‘*Unmasking: the process of self-understanding’* and *‘Impact on relationships’*. This study suggests autistic women’s experiences of self-compassion are generally reduced, and reasons include social challenges, societal expectations and misconceptions of autism. Receiving a diagnosis in adulthood promoted autistic women’s experiences of self-compassion and increased self-understanding.

The study provides insight into the impact of social challenges on autistic women’s experiences of self-compassion. Research suggests some autistic people view ‘difference’ as an experience of not belonging (e.g. Griffith et al., 2012; Ruiz Calzada et al., 2012). Applying Neff’s (2003) ‘isolation over common humanity’ element of self-compassion to the data suggests autistic people’s felt difference is a barrier to developing self-compassion. Participants also commonly described striving traits. It could be argued participants are striving to be accepted by society (as identified in Ward & Meyer, 1999 study) and striving traits may also relate to efforts to accept themselves post-diagnosis. Similarly, Lewis (2016) identified a theme of autistic adults striving to for self-acceptance post-diagnosis.

Participants reported wanting to challenge negative narratives of autism; this is consistent with the Neurodiversity Movement (Silberman, 2017). Autistic self-advocates often challenge the medical model of autism and celebrate autism as inseparable from identity (Baker, 2011; Jaarsma & Welin, 2012; Jordan, 2010). Promoting the voice of the autistic community helps better understand their experience and attempts to counteract the ‘double empathy problem’ (Milton, 2012), which underpins misconceptions of autism. Promoting inclusion in society alongside utilising a participatory approach to research and service development is likely to increase the likelihood that services meet the needs of autistic individuals. In addition, inclusion will reduce autistic individuals’ sense of difference and isolation and facilitate self-compassion in line with Neff’s (2003) common humanity element of self-compassion.

Mental health impact was discussed across the interviews. Mental health issues are associated with lower levels of self-compassion (MacBeth & Gumley, 2012). The common co-occurrence of mental health issues is likely to be a barrier to autistic people’s self-compassion. Research suggested the social challenges associated with autism could increase the risk of mental health issues, including bullying (Bejerot & Mörtberg, 2009; Zablotsky et al., 2014) and non-acceptance from others (Cage et al., 2018; Sasson et al., 2017). Pre-diagnosis participants compared themselves to non-autistic others, feeling different and isolated. Arguably, later diagnosis equates to more years of comparisons and potentially more criticism from others which is internalised as self-criticism. This relates to Neff’s (2003) description of ‘self-judgement’ as a barrier to ‘self-kindness’ and highlights the importance of earlier identification.

Experiences of mental health misdiagnosis were reported; this is consistent with previous research (Bargiela et al., 2016; Eaton, 2018) and is suggested to be underpinned by professionals’ limited understanding of autism (Au-Yeung et al., 2019). Interestingly, Fusar-Poli et al. (2020) reported gender differences in the mental health diagnoses autistic men and women received before their autism diagnosis in adulthood; men were identified to have ‘externalising’ symptoms, for example attention deficit hyperactivity disorder, conduct disorders or psychoses, whereas women received diagnoses with ‘internalising’ behaviours: anxiety, depression or personality disorders. The findings highlight the need for further training for health professionals to reduce misdiagnosis and improve autism awareness.

Participants described societal expectations, feeling different and unhelpful thinking styles as barriers to self-compassion. Research suggests people with low self-compassion are more likely to ruminate and tend to experience extreme emotional responses (Leary et al., 2007; Neff et al., 2007). Research also suggests emotional intensity and rumination positively correlate with autistic traits in autistic and non-autistic people (Brunyé et al., 2012; Joshi et al., 2018; Zhao et al., 2020). This description suggests autistic people are more likely to have reduced self-compassion, compared with non-autistic people. This finding highlights the importance of further research to investigate the effectiveness of therapeutic approaches designed to facilitate self-compassion, for example compassion focused therapy, with autistic individuals.

The diagnosis facilitated autistic women’s experiences of self-compassion and provided permission to assert their needs and self-care activities, including boundaries for social interactions. Participants’ descriptions align with Neff’s (2003) definition of self-compassion, generating self-kindness and understanding towards their difficulties. However, some participants suggested that their diagnosis encouraged more self-analysis, which was not always reported as constructive. Understandably, self-analysis could intensify difficulties, this is the opposite of the mindfulness element of Neff’s (2003) definition of self-compassion, acknowledging suffering without exaggeration.

The potential genetic element of autism was discussed, with many participants reporting family members to have confirmed or suspected autism and described a mutual understanding of each other’s needs. This also demonstrates the interaction between the three flows of compassion (Gilbert, 2010). Recent qualitative research described feelings of closeness and intense connection between autistic mothers and their children with diagnosed or suspected autism (Dugdale et al., 2021). Several participants also reported working with autistic people and having a greater understanding due to their shared diagnosis. This finding supports previous research highlighting the mutual understanding and empathy between autistic people (Komeda, 2015). This finding illustrates the potential benefits of peer support and expert by experience involvement in organisations, particularly mental health services and autism support services.

Misconceptions of autism were reported as a barrier to diagnosis disclosure, particularly with employers. Previous research suggests barriers to autistic people gaining employment included limited understanding of autism, communication difficulties and stigma (Black et al., 2020). This highlights the importance of employers developing autism awareness and inclusion in their organisations to develop connections with autistic individuals and facilitate experiences of self-compassion.

### Future directions

All the participants identified as White, with all but one participant identifying as White British; their experiences may differ from autistic women from other ethnic backgrounds. Simmonds (2021) suggests ‘black autistics wear a triple mask’. It is hypothesised ‘a triple mask’ presents further barriers to self-compassion. A report by the National Autistic Society (Slade, 2014) stated that ethnic minority communities face additional challenges to obtaining autism diagnoses and support. Kelly et al. (2019) reported ethnic minority children had lower levels of autism diagnosis in the United Kingdom. Kandeh et al. (2020) described cultural misunderstanding by service providers and discrepancies between parents and professionals regarding the conceptualisation of autism. Research suggests diagnostic labels including autism may not exist in certain cultures (Bernier et al., 2010; Tincani et al., 2009). In addition, autism stigma and knowledge of autism vary between countries and cultures (Guerrero & Sobotka, 2022; Yu et al., 2020). These findings highlight the importance utilising a participatory approach to increase autism awareness across community groups to end autism stigma. Promoting understanding and acceptance to create more inclusive experiences for all autistic people to facilitate experiences of self-compassion. Future research may wish to explore autistic women’s experiences of diagnosis and self-compassion from other ethnic backgrounds.

Research suggests socio-economic inequalities exist for the diagnosis of autism in the United Kingdom; Kelly et al. (2019) reported that children of mothers with higher education had twice the rate of autism diagnosis, compared to children of mothers with lower-level education status. Information about participants’ SES was not collected, limiting the exploration of the influence of SES on their experiences of diagnosis and self-compassion.

Research exploring the effectiveness of self-compassion-based therapeutic interventions to improve autistic women’s well-being is also recommended.

## Conclusion

Previous research suggests neurotypical women have lower levels of self-compassion than men (Yarnell et al., 2015). This study suggests self-compassion could be even more reduced in autistic women, particularly in those who are undiagnosed. Unhelpful thinking styles, social rejection and misconceptions in relation to autism were commonly reported as barriers to self-compassion. Participants’ felt difference aligns with Neff’s (2003) ‘isolation over common humanity’ highlighting the barriers to self-compassion. This study suggests late diagnosis equates to more years of social challenges, adding to feelings of isolation and detrimental impact on mental health. This emphasises the importance of early identification to reduce the risks associated with low self-compassion and mental health symptoms (MacBeth & Gumley, 2012).

Most participants reported that their diagnosis generated self-kindness and self-understanding, aligning with Neff’s (2003) definition of self-compassion. Participants described the process of adjusting to the diagnosis and learning new ways of self-relating as ongoing, highlighting the importance of post-diagnostic support. For some, the diagnosis also developed compassion towards others, providing a greater understanding of other autistic people’s needs. This included some participants choosing to work with other autistic people, while others considered whether family members might also meet diagnostic criteria, explaining previous interpersonal challenges. Participants also reported increased autonomy post-diagnosis and confidence to assert their needs. Findings from this study suggest that self-compassion interventions could support and enhance autistic women’s well-being.

## Notes

1. Autistic adults and family members favour identity-first terms, for example ‘autistic’ or ‘autistic person’ (Kenny et al., 2016).
